# Predictive Analytics and Workflow Automation to Reduce Administrative Burden and Clinician Burnout in United States Clinical Settings: A Systematic Review

**DOI:** 10.63116/AH.000000010

**Published:** 2026-11-09

**Authors:** Annette Akosah, Thomas J. Hunt

**Affiliations:** Department of Health Informatics, Rutgers School of Health Professions, Rutgers, The State University of New Jersey

**Keywords:** Predictive analytics, Workflow automation, Artificial intelligence, Electronic health records, Administrative burden, Clinician burnout, Ambient AI documentation, Clinical decision support

## Abstract

**Background:**

Electronic health record (EHR)–related administrative burden remains a frequently cited contributor to clinician burnout in the United States. Although digital systems were implemented to improve quality, safety, and care coordination, expanded documentation requirements, inbox messaging, and regulatory reporting have increased clerical workload. Traditional interruptive clinical decision support tools may further increase cognitive demands through alert fatigue and workflow disruption. Health systems have increasingly adopted predictive analytics and workflow-integrated automation, including ambient artificial intelligence (AI) documentation, inbox prioritization, and audit log–based workflow monitoring. However, their effects on operational efficiency and clinician well-being remain uncertain. This review examined clinician-facing interventions in US clinical settings and their impact on documentation workload, operational efficiency, and burnout-related outcomes.

**Methods:**

A systematic review guided by Preferred Reporting Items for Systematic Reviews and Meta-Analyses was conducted using PubMed, CINAHL, and Embase. The search included English-language studies published between January 1, 2019, and December 31, 2025, with an updated search capturing eligible early online publications through 2026. Eligible studies evaluated clinician-facing predictive analytics or workflow automation in US clinical settings and reported measurable outcomes, including documentation time, after-hours EHR use, alert burden, cognitive task load, productivity, or validated burnout measures. Findings were synthesized narratively because interventions and outcome measures varied.

**Results:**

Of 520 records identified, 173 unique records were screened; 24 (13.9%) underwent full-text review, and 7 of 24 (29.2%) met inclusion criteria. Ambient AI documentation platforms were associated with approximately 10%–30% reductions in documentation time, although findings on after-hours EHR activity varied. Several multisite implementations reported improvements in validated burnout measures and cognitive task load. AI-driven inbox prioritization reduced message review time, while audit log–based clinical decision support monitoring identified changes in EHR activity patterns following intervention. Evidence quality varied, and follow-up periods were generally short.

**Conclusions:**

Workflow-integrated predictive analytics and automation interventions were associated with measurable improvements in documentation workload and selected burnout indicators in US clinical settings. However, evidence remains mixed and largely observational. Rigorous, multisite, longitudinal evaluations using standardized workload and burnout metrics are needed to determine sustainability and generalizability.

## Introduction

The widespread adoption of electronic health records (EHRs) has fundamentally reshaped clinical documentation and communication within US health care systems.^1^^,^^2^ While digital transformation was intended to enhance safety, interoperability, and care quality, it has introduced substantial administrative and user complexity. Physicians now devote a significant portion of their workday to electronic documentation tasks, including structured note completion, computerized provider order entry, inbox message management, and regulatory reporting requirements. Time-motion studies have shown that clinicians spend nearly half of their clinical day interacting with the EHR, and for every hour of direct patient care, nearly 2 additional hours may be spent on EHR and desk work.^2^^,^^3^ Audit log analyses likewise demonstrate that documentation frequently continues beyond scheduled clinic hours.^4^

Administrative workload has emerged as a central contributor to clinician burnout, with increasing time devoted to documentation and other clerical tasks in EHR systems.^5^^,^^6^ Burnout, characterized by emotional exhaustion, depersonalization, and reduced professional accomplishment, is associated with lower job satisfaction, increased turnover, and potential patient safety risks.^4^^,^^5^ Documentation burden within EHR systems has also been examined in national technical assessments proposing standardized metrics for measuring clinician workload, including documentation time, after-hours EHR activity, and task switching.^7^ Although burnout is multifactorial, clerical burden and EHR usability consistently rank among the most significant modifiable contributors.^1^

Clinical decision support (CDS) systems were developed to promote evidence-based practice and reduce variability in care delivery.^8^^,^^9^ However, interruptive CDS alerts often introduce additional cognitive demands. Reviews have documented high override rates and alert fatigue, raising concerns that poorly calibrated CDS systems may increase, rather than alleviate, clinician workload.^8^^,^^9^

In recent years, attention has shifted toward workflow-integrated automation strategies designed to reduce manual documentation burden and reorganize administrative tasks within existing clinical workflows. Ambient artificial intelligence (AI) documentation platforms are among the most prominent of these emerging technologies. These systems use speech recognition and natural language processing to capture clinician-patient encounters, generate draft clinical notes, and allow clinicians to review and edit the documentation before finalization in the EHR.^10–12^ Their goal is to improve workflow efficiency, reduce time spent on documentation, and lessen after-hours EHR use.^10^^,^^11^ However, important challenges remain, including concerns related to documentation accuracy, seamless integration into existing EHR systems, data privacy, and the potential for unintended increases in cognitive workload if implementation is poorly managed.^13^^,^^14^ Early evaluations in US health care systems reported reductions in documentation time, decreases in after-hours EHR use, and improvements in cognitive workload and burnout measures.^10^^,^^11^ AI scribes are now used by approximately 30% of physician practices, with adoption continuing to expand across major health systems and EHR platforms.^1^³

Similarly, AI-driven inbox-prioritization tools and predictive audit log–based monitoring systems are designed to streamline clinical task management by identifying high-priority patient messages and analyzing EHR interaction patterns.^15–17^ These approaches leverage machine learning algorithms to classify message urgency and detect patterns in clinician activity, enabling more efficient allocation of attention and time.^15^^,^^16^ However, their effectiveness depends on accurate model performance and seamless integration into clinical workflows. If messages are incorrectly categorized as low priority, delays in review or response may negatively affect patient care and clinical decision-making.^15^ Poorly implemented systems may also contribute to alert fatigue, workflow disruption, or increased cognitive burden.^13^^,^^14^ Unlike traditional interruptive alerts, these tools are intended to support clinician workflow in a more passive and adaptive manner rather than introduce additional interruptions.

## Methods

This systematic review was conducted in accordance with the Preferred Reporting Items for Systematic Reviews and Meta-Analyses 2020 reporting guidelines.^18^ This review synthesizes current evidence on workflow-integrated predictive analytics and automation interventions implemented in US clinical settings, with particular attention to their effects on documentation workload, operational efficiency, and clinician burnout.

A comprehensive literature search was conducted in PubMed, CINAHL, and Embase for studies published between January 1, 2019, and December 31, 2025, with an updated search to capture eligible early online publications through 2026. This time frame was selected to ensure inclusion of the most recent evidence on predictive analytics and workflow-integrated automation while minimizing inclusion of outdated EHR implementations that may not reflect current clinical workflows or technological capabilities. Search terms combined controlled vocabulary and keywords related to ambient artificial intelligence, predictive analytics, workflow automation, CDS, documentation burden, inbox management, and burnout. Notably, all studies meeting final inclusion criteria were published between 2024 and 2026, reflecting the recent and rapidly evolving nature of these technologies in clinical practice.

Studies were eligible for inclusion if they met the following 3 criteria: (1) conducted in US clinical settings; (2) evaluated a clinician-facing predictive analytics or workflow automation intervention; and (3) reported measurable operational, workflow, or workload-related outcomes. Eligible outcomes included documentation time, after-hours EHR activity, alert burden, cognitive task load (eg, NASA Task Load Index), productivity metrics, inbox message volume, or validated burnout measures such as the Professional Fulfillment Index or Mini-Z.

Studies were excluded if conducted outside the US, limited to algorithm development without clinical workflow outcomes, or presented as commentaries, editorials, or narrative reviews without original data. Narrative reviews and other non-original research articles were excluded from the systematic review findings but could be used as background sources to provide context for the Introduction and Discussion. After duplicate removal, titles and abstracts were screened independently. Full-text articles meeting preliminary criteria were reviewed to confirm eligibility. Because of substantial variability in intervention types, outcome measures, and study designs, a quantitative meta-analysis, which is the statistical pooling of results across studies, was not appropriate. Instead, findings were compiled using a narrative approach. The study selection process is illustrated in the Preferred Reporting Items for Systematic Reviews and Meta-Analyses flow diagram (Figure 1).

**Figure 1.**
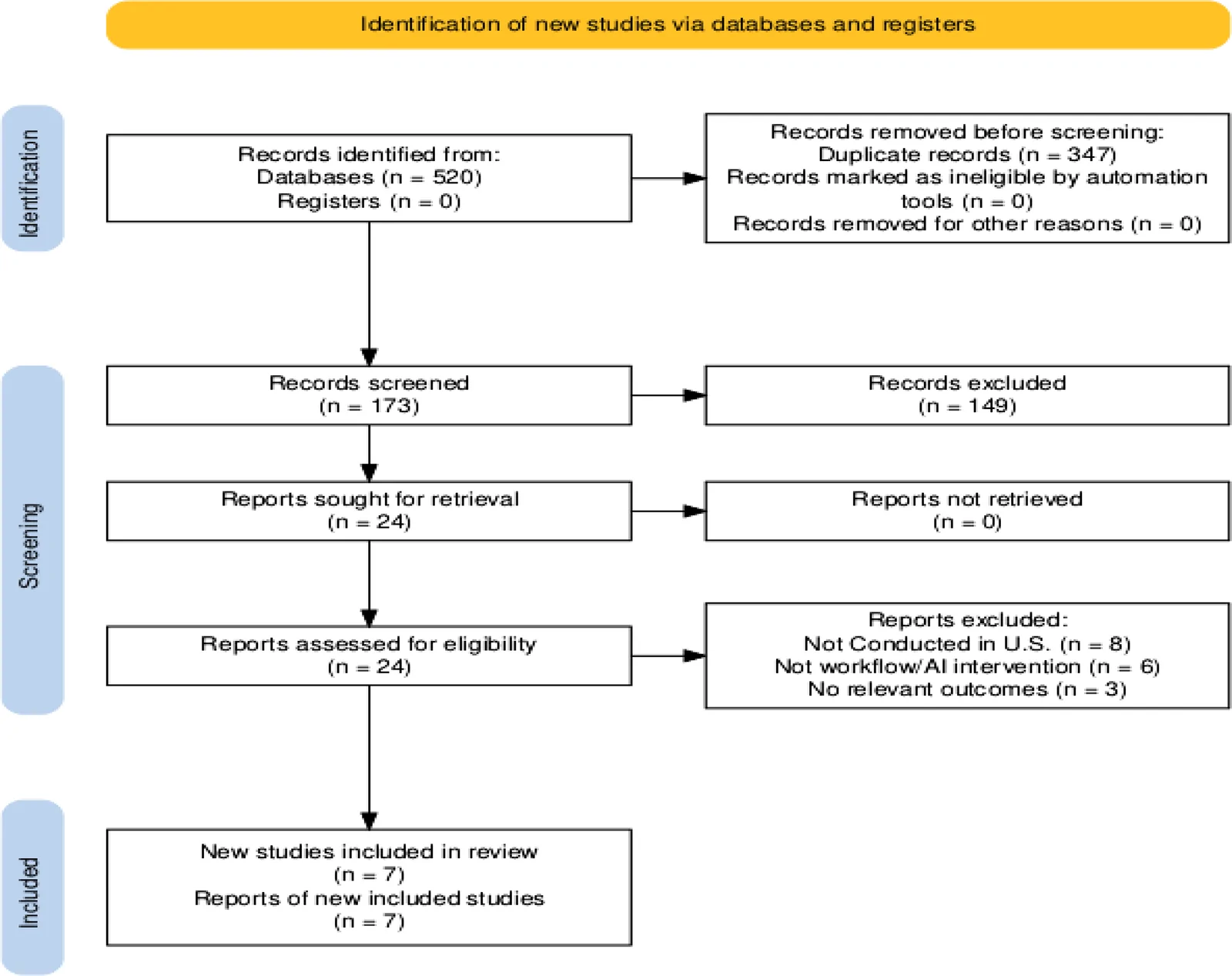
PRISMA Flow Diagram of Study Identification, Screening, Eligibility Assessment, and Inclusion. AI, artificial intelligence; PRISMA, Preferred Reporting Items for Systematic Reviews and Meta-Analyses.

## RESULTS

Seven studies met the inclusion criteria, representing a diverse range of workflow-integrated predictive analytics and automation interventions. Table 1 summarizes the characteristics of included studies, intervention types, study designs, and primary operational outcomes evaluated across the included studies.

**Table 1.** Summary of the Characteristics of Included Studies and Evaluated Interventions

Study	Setting	Intervention type	Study design	Study population/data source	Primary outcomes
Olson et al^10^	Multisite outpatient clinics	Ambient AI documentation	Observational implementation study	Physicians across multiple sites	Documentation time, after-hours EHR use
Stults et al^11^	Ambulatory care	Ambient AI documentation	Implementation study	Clinicians using ambient platform	Documentation burden, workflow measures
Tan et al^12^	Outpatient clinical encounters	Ambient AI scribe	Prospective time-motion study	Clinicians and patients	Documentation time, patient engagement, workflow impact
Haberle et al^19^	Health system	Ambient AI documentation (Nuance Dragon Ambient eXperience [DAX])	Cohort implementation study	Clinicians	Documentation workload, clinician experience
Nguyen et al^15^	Clinical messaging environment	AI-assisted message triage	Observational evaluation	Message dataset	Message review time, prioritization accuracy
Chen et al^16^	CDS environment	CDS audit log workflow analytics	Observational study	CDS audit logs	Workflow pattern changes, task distribution
Holmgren et al^17^	National physician survey	EHR usability and efficiency strategies	Cross-sectional survey	US physicians	Burnout, EHR usability, efficiency strategies

Abbreviations: AI, artificial intelligence; CDS, clinical decision support; EHR, electronic health record.

Most included studies evaluated ambient AI documentation technologies, which represented the largest intervention category. Smaller numbers of studies evaluated AI-assisted inbox triage, CDS-driven workflow monitoring, and broader EHR workflow efficiency strategies (Figure 2).

**Figure 2.**
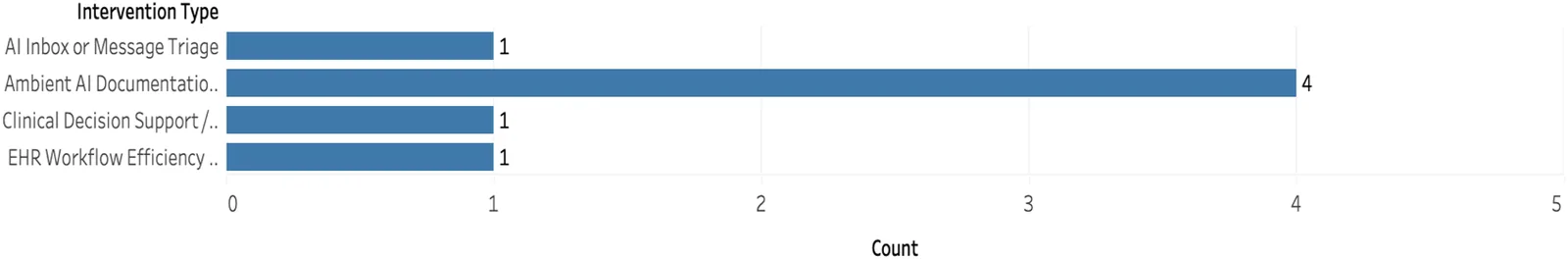
Distribution of Predictive Analytics and Workflow Automation Intervention Types Across Included Studies. AI, artificial intelligence; EHR, electronic health record.

Across multiple health systems and ambulatory settings, ambient AI tools were associated with reductions in time spent documenting per encounter and, in some studies, decreases in after-hours EHR use.^10–12^^,^^19^ Several studies also demonstrated improvements in measures of cognitive workload and clinician experience; however, findings were not uniform across all implementations.^10–12^^,^^19^ In one implementation, improvements in clinician experience and task burden were observed, although the decrease in burnout was not statistically significant.^11^

AI-assisted inbox-prioritization systems demonstrated measurable reductions in message review time and improved identification of high-priority communications.^15^ Although direct burnout outcomes were not consistently reported in inbox-focused studies, operational workflow improvements were evident.

Finally, national survey data examining EHR usability and efficiency strategies demonstrated that the relationship between workflow interventions and burnout may be moderated by baseline system usability.^17^ This finding underscores the contextual nature of workflow interventions.

Across studies, follow-up periods were generally limited to weeks or months, and randomized controlled designs were uncommon, limiting the ability to assess long-term sustainability and causal effects of these interventions. Table 2 summarizes the operational and clinician well-being outcomes reported across the included studies.

**Table 2.** Summary of Reported Operational and Clinician Well-Being Outcomes Across Included Studies

Study	Intervention	Documentation time	After-hours EHR use	Workflow efficiency	Burnout measures
Olson et al^10^	Ambient AI documentation	↓	↓	Improved	Decreased: 51.9% to 38.8%; *P* < .001
Stults et al^11^	Ambient AI documentation	↓	↓	Improved	Decreased: 24 (42.1%) to 20 (35.1%); *P* = .12
Tan et al^12^	Ambient AI scribe	↓	↓	Improved	Not reported
Haberle et al^19^	Ambient AI documentation (Nuance DAX)	↓	↑	Improved	Improved
Nguyen et al^15^	AI-assisted message triage	—	—	↓ message review time	Not reported
Chen et al^16^	CDS workflow analytics	—	—	Workflow pattern changes	Not reported
Holmgren et al^17^	EHR usability and efficiency strategies	—	—	Improved efficiency	Improved

Abbreviations: AI, artificial intelligence; CDS, clinical decision support; DAX, Dragon Ambient eXperience; EHR, electronic health record;↓ indicates a reduction; ↑ indicates an increase; — indicates outcome not reported.

Table 3 provides a study-level synthesis of intervention types, study designs, and the direction of reported operational effects.

**Table 3.** Summary of Study Findings by Intervention Type

Study	Intervention type	Study design	Key operational outcome	Direction of effect
Olson et al^10^	Ambient AI documentation	Multicenter observational	Reduced documentation time and after-hours EHR use	Positive
Stults et al^11^	Ambient AI documentation	Implementation study	Reduced documentation burden and improved workflow efficiency	Positive
Tan et al^12^	Ambient AI scribe	Prospective time-motion study	Reduced documentation time and improved patient interaction	Positive
Haberle et al^19^	Ambient AI documentation (Nuance DAX)	Cohort implementation study	Reduced documentation workload and improved clinician experience	Positive
Nguyen et al^15^	AI-assisted message triage	Observational evaluation	Reduced message review time and improved prioritization accuracy	Positive
Chen et al^16^	CDS workflow analytics	Audit log–based observational study	Shift in task distribution and workflow patterns	Mixed
Holmgren et al^17^	EHR usability and efficiency strategies	Cross-sectional survey	Association between usability improvements and reduced burnout	Mixed

Abbreviations: AI, artificial intelligence; CDS, clinical decision support; EHR, electronic health record.

The operational and clinician workload outcomes evaluated across included studies varied, with documentation time, after-hours EHR use, and clinician experience representing the most frequently reported domains (Figure 3).

**Figure 3.**
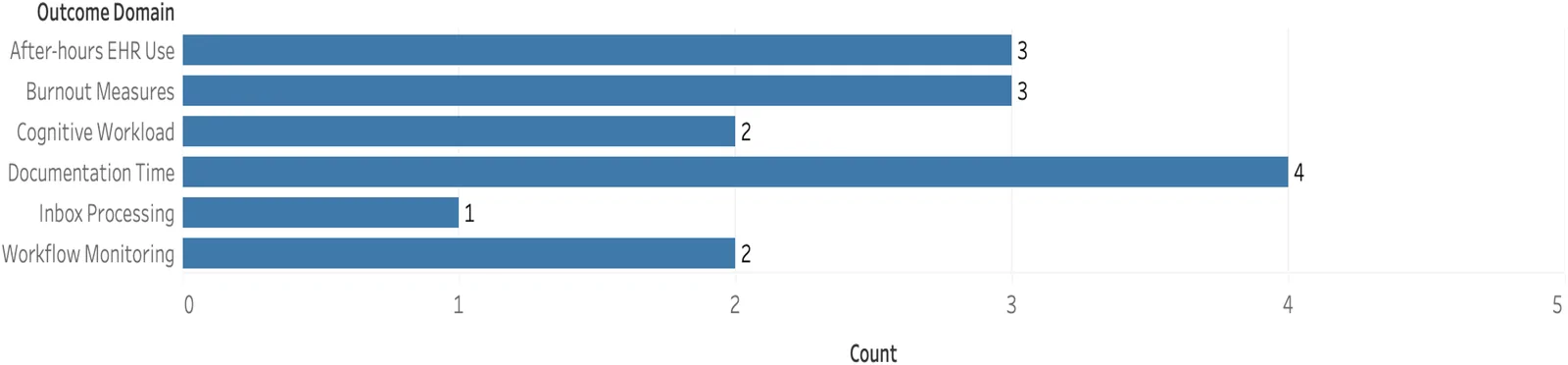
Outcome Domains Evaluated Across Included Studies, Illustrating the Frequency With Which Operational and Clinician Workload Measures Were Reported. EHR, electronic health record.

## Discussion

This review synthesizes emerging evidence that workflow-integrated predictive analytics and automation tools can reduce measurable components of administrative burden in US clinical settings. Ambient AI documentation platforms represented the most frequently studied intervention type and were consistently associated with reductions in documentation time and improvements in aspects of clinician experience.^10–12^ However, findings were not uniform across implementations. Although some studies reported reductions in after-hours EHR use and improvements in workflow efficiency, others reported no significant gains in productivity and, in some studies, after-hours EHR activity increased.^19^

These findings suggest that while ambient AI tools may reduce certain components of documentation burden, the magnitude and consistency of benefit are influenced by implementation context, workflow integration, and baseline system usability. Importantly, efficiency gains do not necessarily address broader concerns related to documentation accuracy, completeness, and clinical reliability. Reports of potential errors, omissions, hallucinations, and misattributed clinical details raise important patient safety concerns.^13^ Reducing physician burden is valuable only if documentation quality, completeness, and clinical decision-making are not compromised in the process. These trade-offs must be carefully evaluated, as ambient AI tools are integrated into routine clinical workflows.

These findings also contrast with historical concerns regarding interruptive CDS systems, which have often increased cognitive load by causing excessive alert fatigue and through workflow disruption.^8^ This contrast underscores a conceptual shift from interruptive to assistive technologies, although both approaches carry potential risks if poorly implemented. Importantly, reductions in documentation time do not uniformly translate into increased productivity or throughput. Evidence from time-motion and implementation studies suggests that gains in efficiency may instead be redirected toward reducing cognitive and clerical burden or improving patient interaction rather than increasing visit volume.^12^^,^^19^ This distinction is critical, as it reframes the value of workflow automation as supporting clinician capacity rather than expanding output.

Improvements in burnout were observed in the selected studies; however, these findings were not consistent across all interventions and were often secondary outcomes.^10^^,^^11^ More commonly, studies reported improvements in related constructs such as task load, satisfaction, or overall clinician experience. Burnout remains influenced by broader structural factors, including staffing levels, organizational culture, reimbursement models, and regulatory requirements.^5^ Cognitive workload frameworks emphasize the importance of aligning digital health technologies with clinician workflows to minimize unintended increases in task complexity and mental demand.^14^ Automation should therefore be viewed as one component of a broader, system-level strategy to support clinician well-being. In this context, the use of audit log analytics to evaluate workflow impact represents a promising methodological advance. These approaches enable near real-time assessment of clinician EHR activity patterns and can help identify unintended consequences of automation and CDS deployment that may not be captured through traditional survey-based measures.^16^

Several limitations of the current evidence should be considered. The majority of included studies were observational or implementation based, limiting the ability to establish causality. Follow-up periods were generally short, restricting insight into the long-term sustainability of observed improvements. In addition, outcome measures varied substantially across studies, particularly in how documentation burden, workflow efficiency, and clinician well-being were defined and measured, limiting comparability and synthesis. Future research should prioritize longitudinal, multisite evaluations using standardized workload and burnout metrics, as well as validated measurement instruments. Such efforts will be essential to determine the durability of intervention effects and to inform scalable, evidence-based implementation strategies within health information management environments.

## Conclusion

Predictive analytics and workflow-integrated automation interventions, including ambient AI documentation systems, AI-assisted inbox prioritization tools, and CDS workflow monitoring, are associated with measurable reductions in documentation workload and improvements in selected indicators of clinician strain in US clinical settings. However, these benefits depend heavily on model reliability. Incorrect classification of urgent patient messages as low priority could delay clinical response and introduce direct patient safety risks. Efficiency improvements must therefore be balanced with strong governance, validation, and clinician oversight.

While early findings are encouraging, the evidence base remains limited in scope and methodological rigor, characterized by predominantly observational study designs, inconsistent outcome measures, and limited long-term follow-up. Longitudinal, multisite evaluations using standardized workload and burnout metrics are needed to determine the durability and generalizability of these interventions. Successful implementation will depend on not only technological capability but also thoughtful workflow integration, usability optimization, strong clinical oversight, and the ability of these systems to generate accurate and clinically reliable documentation. Ongoing governance within health information management environments will be essential to ensure that efficiency gains do not come at the expense of patient safety or documentation quality.

## Conflict of Interest Statement

The authors declare no competing financial interests or conflicts of interest related to this work.

## Funding

The authors received no funding for this research.

## Use of Artificial Intelligence Disclosure

OpenAI ChatGPT (OpenAI; GPT-5) was used on May 4 to assist with formatting guidance, citation formatting review, punctuation, and spelling review during manuscript preparation. All literature screening, study selection, interpretation, synthesis, and final manuscript review were performed and verified by the authors. The authors have nothing to disclose.

CE Quiz
